# Efficacy of total laparoscopic hysterectomy without uterine manipulators in patient with early-stage cervical cancer

**DOI:** 10.12669/pjms.40.10.10093

**Published:** 2024-11

**Authors:** Qiuyan Chen, Qicheng Chen, Haikun Yang, Shifen Dai

**Affiliations:** 1 Qiuyan Chen, Department of Gynecology, Meizhou People’s Hospital, Meizhou, Guangdong Province 514000, P.R. China; 2 Qicheng Chen, Department of Gynecology, Meizhou People’s Hospital, Meizhou, Guangdong Province 514000, P.R. China; 3 Haikun Yang, Department of Gynecology, Meizhou People’s Hospital, Meizhou, Guangdong Province 514000, P.R. China; 4 Shifen Dai, Department of Gynecology, Meizhou People’s Hospital, Meizhou, Guangdong Province 514000, P.R. China

**Keywords:** Total laparoscopic hysterectomy, Uterine manipulator, Early cervical cancer, Feasibility, Safety

## Abstract

**Objective:**

To evaluate the feasibility and safety of total laparoscopic hysterectomy (TLH) without uterine manipulator for patients with early-stage cervical cancer.

**Methods:**

This study was based on retrospective analysis of clinical data of 72 patients with early-stage cervical cancer who received TLH treatment in Meizhou People’s Hospital from January 2018 to December 2020. Of them, 40 patients underwent routine TLH (control group), and 32 patients received TLH without lifting the uterus using uterine manipulator (observation group). Changes in tumor marker levels, human papilloma virus (HPV) status, complications, and survival between two groups of patients were compared.

**Results:**

There was no significant difference in the incidence of postoperative complications between the two groups (*P*>0.05). After the surgery, levels of squamous cell carcinoma antigen (SCC-Ag), carcinoembryonic antigen (CEA), and cancer antigen 125 (CA-125) in both groups of patients were significantly reduced compared to before the surgery, and significantly lower in the observation group compared to the control group at one and two years after the surgery (*P*<0.05). After three years of postoperative follow-up, there was no significant difference in cumulative survival rates between the two groups (*P*>0.05).

**Conclusions:**

Compared with conventional laparoscopy, hysteroscopy without the use of uterine manipulators can significantly reduce the levels of SCC-Ag, CEA, and CA-125 in patients with early-stage cervical cancer within two years after the surgery, without increasing postoperative complications or affecting survival, and has the same safety.

## INTRODUCTION

Cervical cancer is one of the most common malignant tumors in women^1^ with high incidence and mortality, especially in developing countries.^1^,^2^ Total laparoscopic hysterectomy (TLH) remains the main treatment of early cervical cancer, since it is minimally invasive and effective.^3^ However, it has been shown that the conventional TLH using uterine manipulators may be associated with an increased incidence of distant metastasis of cancer cells.^4^ Therefore, a new surgical technique, TLH without lifting the uterus, is becoming more popular.^4^,^5^

This surgical method avoids the use of a uterine manipulator, reduces compression and stimulation of the tumor, and lowers potential transmission of cancer cells during the surgery.^5^,^6^ At present, however, the efficacy and safety of TLH without uterine manipulator is still controversial,^4^,^7^ and conventional TLH remains the mainstay of surgical treatment for patients with early-stage cervical cancer.^8^ This study retrospectively analyzed clinical data of 72 patients with early-stage cervical cancer, aiming to evaluate safety and efficacy of TLH without uterine manipulator in patients with early-stage cervical cancer.

## METHODS

Clinical data from 72 patients with early-stage cervical cancer who underwent TLH in Meizhou People’s Hospital from January 2018 to December 2020 were retrospectively collected. Of them, 40 received conventional TLH treatment and were set as the control group, and 32 patients underwent TLH without the use of uterine manipulator and were assigned to the observation group.

### Ethical approval:

The ethics committee of Meizhou People’s Hospital approved this study with the number 2023-C-90, date: June 2^nd^ 2023.

### Inclusion criteria:

- Women aged between 18 and 70 years old.
- Pathologically diagnosed with stages IB1-IIA2 cervical cancer.^9^
- Complete clinical and pathological data are available for acquisition.

### Exclusion criteria:

- Pregnant or lactating women.
- Patients with serious heart, lung, or kidney diseases.
- History of radiation therapy or chemotherapy.
- Presence of other malignant tumors.
- Presence of severe immune system diseases.
- Severe surgical contraindications, such as abnormal coagulation function.

### Standard TLH^10^:

The patient underwent bladder lithotomy under general anesthesia in the operating room, followed by routine disinfection and drape, indwelling a urinary catheter, and fixing the uterus with a uterine manipulator. A 1cm incision was made above the navel, pneumoperitoneum was punctured and Trocar (diameter 1cm) inserted. Pneumoperitoneum pressure of 12mmHg was maintained, and the abdominal cavity was inflated at a flow rate of 20L/minutes. Laparoscopic insertion and exploration were done for the adhesion between the sigmoid colon and the left pelvic wall, liver, gallbladder, spleen, stomach, intestinal tract, subphrenic, omental surface, uterine size, surface. Skin incisions of approximately 0.5cm long were done at the left and right Macquard’s points and above the left and right pubic symphysis, and Trocar (diameter 0.5cm) was inserted. We then performed extensive hysterectomy, double adnexectomy, and pelvic lymph node dissection.

### TLH without uterine manipulator:

TLH without uterine manipulator was first described by Kavallaris et al.^11^ Surgical techniques of TLH without uterine manipulator in this study followed the description of previous studies.^10^-^12^ Non-invasive forceps were used instead of uterine manipulator to clamp both sides of the uterine horns to assist in exposing the surgical field of view. When cutting off the vagina in a closed manner, the cerclage technique was used to seal the tumor body, and the absorbable thread No. 1 was used to perform cerclage 3cm below the vaginal fornix. Vaginal wall was then cut off 1cm below the ligation line. The excised lymph nodes were immediately placed in the specimen strip, and after removing the detached tissue, the vagina and pelvic surgical area were rinsed with a large amount of physiological saline. At the end of the surgery, pelvic and abdominal cavities were thoroughly rinsed with sterilized physiological saline, vaginal stump and vaginal wall disinfected and cleaned.

### Observation indicators:

Levels of tumor markers, including SCC-Ag, CEA, and CA125, were measured in the serum of fasting venous blood using Roche Elecsys-2010 fully automatic electrochemiluminescence immunoassay analyzer and corresponding reagent kit for measurement.

The occurrence of complications, such as injury, bleeding, infection, thrombosis, and lymphedema/cyst.

### Mortality:

patients were followed-up for three years after the surgery to assess survival status.

### Statistical analysis:

Data were analyzed using SPSS version 26.0 (IBM Corp, Armonk, NY, USA). Quantitative data were represented by mean ± standard deviation, independent sample *t*-test was used for inter group comparison, and paired *t*-test was used for intra group before and after comparison. Count data were analyzed using chi square test to represent the number of use cases. The cumulative survival rate of patients at three years after the surgery was calculated using Kaplan Meier method. *P*<0.05 indicated statistically significant difference. PRISM 8.0 software (GraphPad, San Diego, USA) was used to plot the postoperative changes in SCC-Ag, CEA, and CA125 levels in patients.

## RESULTS

**Fig.1:**
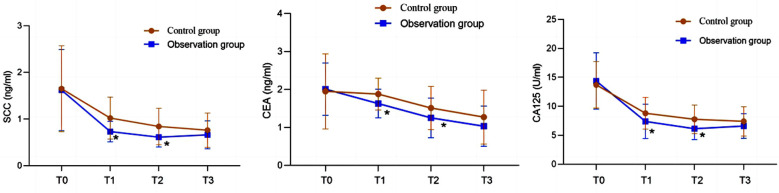
SCC-Ag, CEA, CA125 level change curve chart; Compared with the control group, * P<0.05. T0: preoperative; T1: one year after surgery; T2: two years after surgery; T3: three years after surgery.

**Fig.2:**
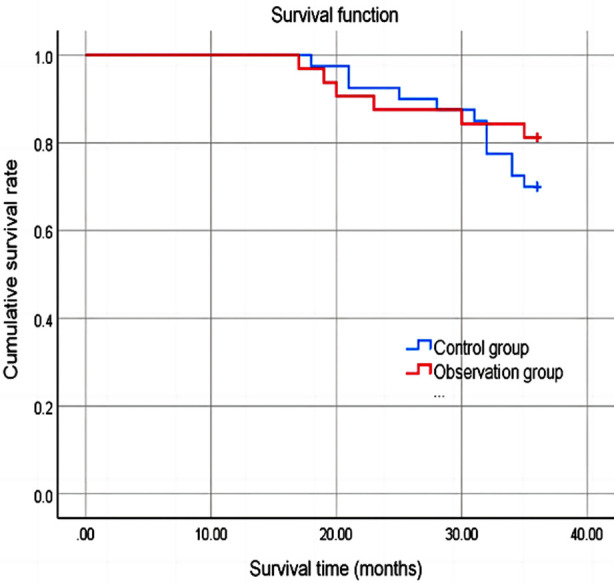
Three-year postoperative survival rate.

A total of 72 patients met the conditions for this study. Age ranged from 29 to 67 years old, with an average of 48.63 ± 8.18 years. There were 40 cases in the control group and 32 cases in the observation group, with no significant difference in baseline data between the two groups (*P*>0.05) (Table-I). After the surgery, there was no significant difference in the incidence of complications between the two groups (*P*>0.05) (Table-II). Before the surgery, there was no significant difference in the levels of SCC-Ag, CEA, and CA125 between the two groups (*P*>0.05). After surgery, the levels of SCC-Ag, CEA, and CA125 in both groups were significantly lower than before, and at one and two years after surgery, and significantly lower in the observation group compared to the control group (*P*<0.05) Fig.1. Three years after the surgery, no recurrence or metastases were observed. In the control group, four cases of recurrence or metastases were found, but with no significant difference between the two groups (*P*>0.05). After three years of postoperative follow-up, there was no significant difference in cumulative survival rates between the two groups (*P*>0.05) (Fig.2).

**Table-I:** Comparison of clinical data between the two groups.

Item	Category	Control group (n=40)	Observation group (n=32)	t/χ^2^	P
Age (years)		48.90±8.73	48.28±7.56	0.317	0.752
Pathological type	Squamous cell carcinoma	28 (70.00)	28 (87.50)	5.580	0.061
	Adenocarcinoma	12 (30.00)	3 (9.40)		
	Adenosquamous carcinoma	0 (0.00)	1 (3.10)		
Tumor grading	G1	2 (5.00)	2 (6.25)	0.180	0.914
	G2	34 (85.00)	26 (81.25)		
	G3	4 (10.00)	4 (12.50)		
Tumor Size (cm)		3.25±1.24	3.37±1.50	-0.386	0.701

**Table-II:** Comparison of postoperative complications between the two groups.

Group	Injure	Hemorrhage	Infect	Thrombus	Lymphedema /cyst	Overall complication rate (%)
Control group (n=40)	0 (0.00)	0 (0.00)	7 (17.50)	0 (0.00)	2 (5.00)	9 (22.50)
Observation group (n=32)	0 (0.00)	0 (0.00)	6 (18.75)	0 (0.00)	0 (0.00)	6 (18.75)
*χ^2^*						1.647
*P*						0.439

## DISCUSSION

The results of this study showed that TLH without uterine manipulator is equally safe as the standard TLH, and is not associated with the increased incidence of complications. The results basically in line with Zeng et al.^10^ TLH without uterine manipulator avoids the use of manipulator cups, which may reduce damage to tissues and structures, thus lowering the risk of injury, bleeding, and infection.^7^,^13^ Moreover, this method minimizes pressure on the tumor during the surgery, preventing dissemination of tumor cells and reducing complications caused by tumor rupture or injury.^7^,^14^

Chen et al.^15^ used a closed vaginal incision to treat early stage cervical cancer without the use of uterine manipulators, and reported quick recovery of patients. The study by Yuan et al.^16^ also showed that not using uterine manipulators is efficient for treating early cervical cancer, with no complications. In addition, Zygouris et al.^12^ retrospectively analyzed 1023 patients who underwent TLH without using any type of uterine manipulator, and showed that the surgery was safe and feasible, which is consistent with our results.

This study also found that TLH without uterine manipulator was associated with lower levels of SCC-Ag, CEA, and CA125. Levels of SCC-Ag, CEA, and CA125 can reflect size, staging, and prognosis of tumors, and can be used to monitor treatment efficacy and recurrence.^17^,^18^ Our results demonstrate that TLH without uterine manipulator may have a positive impact on the efficacy of surgical treatment. Lack of significant difference in survival between the two groups of patients suggests that TLH without uterine manipulator does not compromise the prognosis, which is consistent with previous observations.^19^-^21^

Mebes et al.^22^ found that TLH without uterine manipulator can reduce postoperative pain and discomfort in patients, thereby improving their quality of life, and that this surgical method was associated with a lower recurrence rate, as indicated by the long-term follow-up data. However, further studies are needed to gain a deeper understanding of the mechanisms behind this effect.

### Limitations:

It is a single-center retrospective study. Firstly, the sample size is small and there are few observation indicators, especially perioperative indicators. Secondly, the postoperative follow-up period was only three years. Longer follow-up is needed to verify our results. Finally, the impact of the two surgical methods on the long-term functional recovery of patients was not analyzed. Therefore, more prospective, multicenter clinical studies are needed to validate our results and further evaluate the clinical efficacy and safety of TLH without uterine manipulator in the treatment of early cervical cancer.

## CONCLUSION

Compared with conventional TLH, TLH without uterine manipulator can significantly reduce the levels of SCC-Ag, CEA, and CA125 in patients with early-stage cervical cancer within three years after surgery, without increasing postoperative complications or affecting survival, and has the same safety.

### Authors’ contributions:

**QC:** Conceived and designed the study, Review. **QC, HY and SD:** Collected the data and performed the analysis. **QC:** Was involved in the writing of the manuscript and is responsible for the integrity of the study. All authors have read and approved the final manuscript.
